# Knowledge, Attitudes, Anxiety, and Preventive Behaviors Related to COVID-19 Among Healthcare Providers: A Developing Country’s Perspective

**DOI:** 10.7759/cureus.51026

**Published:** 2023-12-24

**Authors:** Muhammad Hammad, Sadaf Fardoos, Rasikh Arif, Usman Ghani, Shailes Paudel, Krishna Vardhan, Hima Bindu Reddy Basani, Sreehitha Challa, Ali Zeb Khan, Altamash Dad Khan

**Affiliations:** 1 Pharmacy, Shifa College of Pharmaceutical Sciences, Shifa Tameer-E-Millat University, Islamabad, PAK; 2 Pharmacy, Shifa College of Medicine, Shifa Tameer-E-Millat University, Islamabad, PAK; 3 Healthcare Management, School of Business and Management, Riphah International University, Islamabad, PAK; 4 Pharmacy, Riphah Institute of Pharmaceutical Sciences, Riphah International University, Islamabad, PAK; 5 Research Department, Al-Shifa Research Centre, Al-Shifa Trust Eye Hospital, Rawalpindi, PAK; 6 Clinical Research, Aga Khan University Hospital, Karachi, PAK; 7 Intensive Care Unit, Patan Academy of Health Sciences, Lalitpur, NPL; 8 General Medicine, Indira Gandhi Medical College and Research Institute, Puducherry, IND; 9 Graduate Medical Education, Lyceum Northwestern University, Dagupan City, PHL; 10 School of Public Health, University of Texas Health Science Centre, Houston, USA; 11 Graduate Medical Education, Southwestern University, Cebu, PHL; 12 Department of Biotechnology, University of Karachi, Karachi, PAK

**Keywords:** anxiety, attitudes, covid-19, healthcare workers (hcw's), knowledge, pakistan, preventive behaviors

## Abstract

Background

In early 2020, the rapid global spread of coronavirus disease 2019 (COVID-19) presented healthcare workers (HCWs) with an unprecedented challenge. The constant influx of new information about the virus created knowledge gaps, and the relentless demands of their work schedules left many feeling overwhelmed. This paper explores the knowledge, attitudes, anxiety levels, and preventive behaviors of HCWs in the twin cities of Pakistan, Rawalpindi and Islamabad, related to the COVID-19 pandemic. In the face of this crisis, it is essential to gain insights into the experiences and needs of these frontline heroes.

Methodology

We conducted a descriptive web-based cross-sectional study among healthcare professionals to delve into the multifaceted aspects of their experiences. This included analyzing sociodemographic characteristics, knowledge levels, attitudes, practices, and the psychological implications of the pandemic. By employing both quantitative and qualitative analysis, we aimed to provide a comprehensive understanding of these parameters.

Results

Out of the 1150 responses collected, it became evident that breathing problems were the most recognized symptom of COVID-19 among HCWs. Impressively, 95.7% of participants believed in the primarily symptomatic nature of treatment, although 37% held the misconception that antibiotics were effective against the virus. Another noteworthy discovery was that 68.2% of HCWs favored testing before initiating treatment, yet a concerning 22.7% of treated patients received no testing. A significant proportion, approximately 19.6%, experienced heightened anxiety levels during the pandemic, leading to an increased frequency of handwashing. In terms of preventive behaviors, a majority of respondents displayed a heightened sense of caution. Nearly 59.1% reported avoiding the use of others' accessories and emphasized not reusing face masks. Furthermore, 84.1% of participants preferred staying at home during quarantine.

Conclusion

The study reveals the profound impact of COVID-19 on healthcare providers in Pakistan's twin cities, highlighting elevated anxiety levels among most. This underscores the urgent need for mental health support. Beyond physical effects, the pandemic significantly strains their psychological well-being. To address this stress, vital strategies include workload reduction, structured shifts, stressor minimization, and improved knowledge sharing. Cultivating a healthy work environment is equally crucial for healthcare providers' overall well-being. These insights are essential for crafting policies and interventions to better protect and support frontline workers in a developing nation like Pakistan, demonstrating healthcare professionals' resilience and dedication amid unprecedented challenges.

## Introduction

In late 2019, an obscure yet formidable adversary emerged on the global stage, the coronavirus disease 2019 (COVID-19) pandemic. The World Health Organization (WHO) christened it as such, and this viral interloper swiftly unleashed a pneumonia outbreak in the heart of Wuhan City, Hubei Province, China [1]. This infectious scourge was traced back to a β-coronavirus, known as the severe acute respiratory syndrome-related coronavirus 2 (SARS-CoV-2), characterized by its enveloped non-segmented positive-sense RNA virus nature. The world, unaware of the impending challenges, was thrust into a new era of health crises, leaving us no choice but to adapt and overcome them [2].

This novel adversary, COVID-19, demonstrates remarkable contagiousness, primarily propagating from one individual to another within close proximity, typically defined as a mere six feet. It harnesses respiratory droplets as its stealthy vehicle, silently transferring its genetic cargo through coughs, sneezes, and even insidious contact with contaminated objects. In the blink of an eye, it infiltrates through the portals of our eyes, nose, and mouth, reminding us of our shared vulnerability [3].

As of May 10, 2023, the world has collectively borne the weight of 765,903,278 confirmed COVID-19 cases and mourned the loss of 6,927,378 souls, as reported to the vigilant custodians at WHO. The echoes of this relentless pandemic reverberate in every corner of our planet, reminding us of our shared responsibility to protect and heal [4].

In Pakistan, the first case of COVID-19 cast its ominous shadow on February 26, 2020. Since then, the nation has faced 1,580,631 confirmed cases, with 30,656 souls lost to the relentless grip of this virus. Despite the initial onslaught, hope emerges as a beacon of light; the number of cases has been slowly receding since the dawn of 2023, with a remarkable recovery rate of 98.06% as of April 26, 2023. Our resilience is a testament to human fortitude [5].

Front and center in this battle are the Healthcare Workers (HCWs). These valiant souls stand as the vanguard against COVID-19, shouldering the immense responsibility of care [6]. Yet, they are also the most susceptible to the virus's clutches. The preventive measures they employ are not just for their well-being but for the collective defense against the relentless pandemic [7]. Their expertise, perspective, and unwavering commitment are the linchpins in the containment of this highly contagious illness [8].

In Pakistan, the onslaught of COVID-19 had a disproportionate impact on the healthcare system [9]. The toll on the frontline HCWs was profound, as infections spread like wildfire. It led to a painful exodus of healthcare professionals, which further strained the already fragile healthcare system [10]. Their struggle goes beyond physical health; it's a battle for mental well-being, combating the shadows of despair cast by a virulent foe. Moreover, the specter of brain drain has haunted Pakistani HCWs, undermining effective healthcare delivery [11].

Drawing inspiration from a study in Yemen, where healthcare workers displayed commendable knowledge 69.8%, an optimistic attitude 85.10%, and a moderate level of anxiety 51.0% towards COVID-19, our own study seeks to illuminate the knowledge, attitudes, anxiety, and preventive behaviors of HCWs in Pakistan regarding this formidable adversary [12]. Through this inquiry, we aim to craft a blueprint for improved treatment policies and effective regimens. With interventions that encompass knowledge dissemination and stress management, we aspire to bolster our healthcare providers, the unsung heroes in this saga, who work tirelessly to heal and protect our society.

In the face of COVID-19, we stand at the precipice of transformation. Our understanding, our attitudes, and our resilience are the currency that will carry us through. Together, as a global community, we strive for a future where the specter of pandemics does not overshadow our shared humanity but instead strengthens our resolve to protect, heal, and uplift one another.

## Materials and methods

A cross-sectional survey was conducted in Pakistan's twin cities, Rawalpindi and Islamabad, using an online questionnaire to gather insights from healthcare providers. Despite the challenges posed by the pandemic, the study adapted ingeniously by adopting a digital approach while adhering to pandemic protocols. The survey was conducted from March 2021 to April 2021, providing a timely snapshot of knowledge, attitudes, anxiety, and preventive behaviors among this critical demographic.

Study instrument

To unravel the complex impact of COVID-19 on healthcare providers in a developing nation, we utilized modern technology and survey design. We drew inspiration from prior research [12] and constructed a questionnaire as our research tool. This digital instrument, comprising 40 questions (see Appendix), represented a blend of rigor and innovation. Instead of being a static document, it took the form of a dynamic Google Form accessible through a web portal, inviting responses from healthcare professionals on the front. The questionnaire was meticulously structured, incorporating both closed-ended queries and open-ended exploratory questions. It unfolded in six distinct sections, each with a unique purpose. The first section focused on identifying respondents through email addresses and date stamps. The second section delved into demographics, including gender, age, and medical expertise. Moving deeper into the realms of knowledge and awareness, the third section featured 10 questions designed to assess respondents' understanding of COVID-19. Sections four and five explored attitudes and anxiety with six and 16 inquiries, respectively. Finally, the closing section centered on frontline experiences, inquiring about the specific preventive measures taken by healthcare providers and their perspectives on mental health mobile applications through an additional six questions. This questionnaire acted as a conduit for insights, offering response options of "YES," "NO," or "DON'T KNOW." These binary and nuanced responses allowed us to grasp the extent of knowledge, anxiety levels, attitudes, and preventive behaviors exhibited by healthcare heroes in the face of the COVID-19 challenge.

The analytical journey, driven by the digital age, transcended mere statistical analysis. It was an immersive exploration of the human experience during a pandemic. We employed descriptive statistics, incorporating frequencies and percentages to reveal the intricate sociodemographic characteristics, knowledge, attitudes, anxiety levels, and preventive behaviour implications hidden within our survey using SPSS software, version 26.0 (IBM Corp., Armonk, NY). Age frequencies served as our guide, helping us distinguish between seasoned veterans and newcomers. In other sections, we encouraged descriptive responses to capture the essence of participants' thoughts about this viral journey. Through this innovative approach, our aim was not only to quantify but to genuinely understand the experiences of healthcare providers in their battle against COVID-19.

Data collection

Amid the relentless pandemic in our twin cities, safeguarding the well-being of our dedicated healthcare professionals became paramount. As traditional avenues for direct access to healthcare providers in medical facilities dwindled, we ventured into the digital realm to overcome this challenge. Our tool of choice was an innovative online data collection method using the versatile Google Forms platform. Our data collection effort targeted four categories of healthcare professionals: physicians, pharmacists, paramedical staff, and medical lab technologists. To ensure clarity and significance, we used telecommunications to explain the questionnaire's purpose and importance. We meticulously crafted a web-based self-report electronic questionnaire, aligning it with established COVID-19 guidelines. This tool aimed to uncover insights into the experiences, perspectives, and challenges faced by our healthcare heroes. Our pursuit of participation knew no bounds as we cast a wide net through the digital landscape. Links to the questionnaire circulated in email inboxes echoed in WhatsApp groups and resonated across various social media platforms. Our inclusive approach transcended gender and geography, welcoming all healthcare professionals in the twin cities aged 20 years or above with a basic understanding of COVID-19. In this digital journey, our comprehensive online data collection approach unveiled a tapestry of insights, enriching our understanding of the pandemic's impact and the challenges confronting our healthcare system. In this synergy of technology and human dedication, we illuminate the path forward.

## Results

Our research embarks on an illuminating journey, delving into the intricate tapestry of how healthcare professionals are navigating the uncharted waters of the global pandemic. In this dynamic study, we unveil a vivid and ever-evolving spectrum of responses from healthcare providers within a developing nation. As we peel back the layers of our findings, we uncover the nuanced experiences and perspectives that each gender cohort, predominantly comprising 78.3% females and 21.7% males, brings to the forefront in the battle against the formidable adversary known as COVID-19 (Table 1).

**Table 1:** Demographic characteristics of the HCWs HCWs: Healthcare workers.

Variables	Frequency (n)	Percentage (%)
Gender		
Male	249	21.7
Female	901	78.3
Age (years)		
23-27	940	81.7
28-32	131	11.4
33-37	26	2.3
38-42	53	4.6
43 and above	0	0
Health Care Domain		
Physician	510	44.35
Pharmacist	357	31.04
Nurse	179	15.56
Paramedical Staff	104	9.05
Medical Lab Technologist	0	0
Work Experience		
<1 month	307	26.7
1 month but <3 months	77	6.7
3 months but <12 months	77	6.7
1 year but <3 years	383	33.3
1 year but <3 years	255	22.2
≥ 10 years	51	4.4
Locality		
Urban	949	82.5
Rural	201	17.5

Shifting our gaze towards the canvas of age demographics, our data paints a captivating portrait. A staggering 81.7% of the healthcare workforce falls within the age bracket of 23-27 years, a testament to the youthful dynamism propelling the frontline. However, within this mosaic, we also acknowledge the substantial presence of healthcare providers aged 43 years and older, individuals who contribute a wealth of experience and wisdom to the ongoing struggle against the viral onslaught.

Navigating through the diverse terrain of healthcare domains, our study unveils physicians, pharmacists, and nurses as the dominant players, comprising 44.35%, 31.04%, and 15.56%, respectively. Yet, we must not overlook the indispensable roles played by paramedical staff and medical lab technologists, each contributing 9.05% and 0%, respectively, showcasing the symphony of skills and expertise within this collective effort. Lastly, when we examine work experience, it is striking to note that a notable 26.7% of our participants possess less than one month of experience, underscoring the urgency and rapid enlistment of new healthcare providers into the pandemic's frontline battalions.

Our research proceeds to unveil a comprehensive tableau of responses from healthcare workers regarding their knowledge, attitudes, anxieties, and preventive behaviors related to COVID-19. Table 2 stands as a testament to their collective wisdom. Remarkably, over 80% of healthcare providers exhibit accurate identification of common symptoms such as cough and fever. However, disparities emerge when recognizing less prevalent symptoms like diarrhea and myalgia, underscoring the imperative need for more comprehensive training and education.

**Table 2:** The responses of HCWs with regard to their knowledge, attitude, anxiety, and preventive behaviors related to COVID-19 HCWs: Healthcare workers; COVID-19: Coronavirus disease 2019.

Variables		Percentage (%)
Knowledge towards COVID-19		
The main clinical symptoms of COVID-19 are: (Tick all that apply)	Cough	82.6
	Fever	84.8
	Sore throat	69.6
	Runny nose	34.8
	Myalgia	47.8
	Diarrhoea	30.4
	Difficulty in breathing	93.5
Loss of taste and smell are also the feature of COVID-19 infection:	Yes	93.5
	No	0.4
	Don’t Know	6.1
There is currently no effective cure for COVID-19, but only symptomatic and supportive treatment can help most patients recover from the infection:	Yes	95.7
	No	4.3
	Don’t Know	0.0
Are antibiotics effective against coronavirus?	Yes	37.0
	No	56.5
	Don’t Know	6.5
It is not necessary for children and adults to take measures to prevent the infection by the covid-19 virus:	Yes	97.1
	No	0.0
	Don’t Know	2.9
Persons with COVID-19 cannot transmit the virus to others when fever is not present:	Yes	10.9
	No	87.0
	Don’t Know	2.1
Covid-19 virus spreads via respiratory droplets:	Yes	95.7
	No	0.0
	Don’t Know	4.3
It takes 14 days for corona virus symptoms to appear:	Yes	56.5
	No	43.5
	Don’t Know	0.0
Majority of COVID-19 infective patient will not develop illness but elderly patient having chronic illness, DM, COPD, PNEUMONIA is likely to develop severe illness.	Yes	84.8
	No	10.9
	Don’t Know	4.3
Health care professional with direct contact should take tablet hydroxychloroquine as a prophylaxis:	Yes	39.1
	No	43.5
	Don’t Know	17.4
Attitude towards COVID-19		
Are you currently able to test your patients for COVID-19 quickly and easily?	Yes	59.1
	No	38.6
	Don’t Know	2.3
How many patients have you treated with possible COVID-19 symptoms, but have not been able to test for COVID-19?	0-5 patients	68.2
	6-10 patients	6.7
	11-15 patients	2.4
	More than 15	22.7
Are you concerned about patients avoiding testing or treatment due to financial or health insurance barriers?	Yes	93.2
	No	5.4
	Don’t Know	1.4
Has the government taken appropriate measures to support the medical supply chain and ensure that your hospital/clinic has the medical supplies necessary to address the COVID- 19 pandemic?	Yes	57.8
	No	35.6
	Don’t Know	6.6
In response to COVID-19, have you increased your use of telemedicine technologies in your clinical practice?	Yes, I have started seeing patients virtually	41.9
	No, we have not	39.5
	We’re planning to	18.6
Among elderly, children, immuno-compromised and adults who do you think exhibit a high complication rate? (Tick all that apply)	Elderly	26.7
	Children	56.8
	Adults	6.6
	Immune-compromised individuals	66.7
Anxiety towards COVID-19		
How frequently do you wash your hands?	Every 15 minutes and when required	6.5
	Every 30 minutes and when required	19.6
	Every 1 hour and when required	15.2
	As and when required	58.7
Do you feel anxious in gatherings due to COVID-19?	Yes	80.4
	No	19.6
	Don’t Know	0.0
Do you think that following COVID-19 SOP’s is leading to OCD (Obsessive Compulsive Disorder)?	Yes	45.7
	No	50.0
	Don’t Know	4.3
Do you take antidepressants due to this pandemic situation?	Yes	8.7
	No	87.0
	Don’t Know	4.3
In this pandemic, do health care professionals need mental assistance:	Yes	77.8
	No	15.6
	Don’t Know	6.6
Are you anxious about following patients with COVID‐19?	Yes	80.0
	No	15.6
	Don’t Know	4.4
Worried about being infected?	Yes	57.8
	No	35.6
	Don’t Know	6.6
Strict self-protection against covid-19?	Yes	76.1
	No	19.6
	Don’t Know	4.3
Does COVID-19 have impact on your personal life?	Yes	73.9
	No	23.9
	Don’t Know	2.2
Have you been ostracized?	Yes	23.9
	No	60.9
	Don’t Know	15.2
Due to long working hours and attending ample of patients, do you feel depressed/anxious?	Yes	46.7
	No	40.0
	Don’t Know	13.3
Do you feel anxious in following all SOP’s around COVID-19 patients?	Yes	53.3
	No	42.2
	Don’t Know	4.5
Do you have fear of lack of medication and uncontrolled viral spread?	Yes	74.4
	No	20.9
	Don’t Know	4.7
Are you anxious in counselling patients against non-compliance to the COVID-19 vaccine?	Yes	63.6
	No	29.5
	Don’t Know	6.9
Does scarcity of ventilators give you depression?	Yes	62.2
	No	31.1
	Don’t Know	6.7
Preventive Behaviors against COVID-19		
Have you been practicing social distancing?	Yes	88.6
	No	9.1
	Don’t Know	2.3
Do you use other workers’ phones, desks and tools?	Yes	46.5
	No	51.2
	Don’t Know	2.3
Do you reuse a mask?	Yes	38.6
	No	59.1
	Don’t Know	2.3
Do you prefer to stay at home during the quarantine?	Yes	84.1
	No	13.6
	Don’t Know	2.3

A substantial majority, amounting to 93.5%, correctly associates the loss of taste and smell with COVID-19, a critical diagnostic indicator. Almost the entirety of our respondents, totaling 95.7%, understand the stark reality that there exists no known cure for COVID-19, emphasizing the paramount importance of symptom management and prevention.

Notably, 56.5% of respondents correctly acknowledge the ineffectiveness of antibiotics against the coronavirus, signaling a need for education on appropriate treatments. Encouragingly, 97.1% recognize the necessity of preventive measures for all age groups, highlighting the pivotal role of healthcare providers in disseminating this life-saving information. The majority, accounting for 87.0%, correctly understand that COVID-19 can be transmitted without the presence of fever, emphasizing the need for vigilance in asymptomatic cases. Moreover, most respondents, totaling 95.7%, accurately identify respiratory droplets as the primary mode of COVID-19 transmission, underscoring the critical importance of mask-wearing and adherence to social distancing measures. Knowledge about the incubation period is somewhat divided, with 56.5% being aware of the 14-day timeframe, indicating the necessity for increased awareness to effectively monitor and manage potential cases. Further, the majority, amounting to 84.8%, correctly identifies elderly patients with chronic illnesses as particularly susceptible to severe illness, signaling an understanding of the vulnerable populations at stake. Turning our lens towards prophylaxis, a degree of uncertainty prevails, with 43.5% of healthcare professionals expressing uncertainty about the use of hydroxychloroquine as a preventive measure. This uncertainty underscores the need for clearer guidelines in this realm.

The attitudes of healthcare providers are pivotal factors that significantly influence patient care and public health outcomes. Notably, 59.1% of respondents report the capability to swiftly conduct COVID-19 tests, while 38.6% face limitations in accessibility to testing, thereby illuminating a pressing concern. A substantial majority, representing 68.2%, finds themselves treating patients with COVID-19 symptoms without the benefit of access to testing, highlighting potential diagnostic challenges. Concerns surrounding patients avoiding testing due to financial or insurance barriers loom large, with 93.2% expressing apprehensions, suggesting the imperative need for policy interventions to address this barrier. In terms of government support, a moderate number, constituting 57.8%, believe that governments have provided adequate support to the medical supply chain, signifying room for potential improvement in this regard. On the telemedicine front, 41.9% have adopted this mode of care delivery, showcasing the adaptability of healthcare providers in response to the evolving demands of the pandemic. Opinions on complication rates vary, with healthcare providers differing in their perspectives on who is most at risk.

The mental well-being of healthcare providers emerges as a critical facet of this study. An encouraging 58.7% exhibit commendable adherence to hand hygiene protocols, reflecting an elevated sense of personal hygiene. Nevertheless, the majority, totaling 80.4%, experiences heightened anxiety in social gatherings due to COVID-19, underscoring the profound psychological toll exacted by the pandemic. A significant portion, amounting to 45.7%, believes that strict adherence to COVID-19 standard operating procedures might lead to symptoms resembling Obsessive Compulsive Disorder (OCD), suggesting potential mental health concerns within the healthcare community. It is noteworthy that a minority, accounting for 8.7%, relies on antidepressants, hinting at the need for targeted mental health support among certain healthcare providers. Furthermore, a significant proportion, totaling 77.8%, expresses the belief that healthcare professionals require mental assistance, further highlighting the profound impact of the pandemic on their well-being. Anxiety levels are notably elevated concerning patient follow-up, personal safety, self-protection, and the broader ramifications of COVID-19 on their personal lives and professions.

The actions of healthcare providers reverberate as a powerful model for the broader community. Our study reveals that an overwhelming majority, comprising 88.6%, actively practices social distancing, setting a resounding example for others to follow. Despite elevated anxiety levels, hand hygiene remains a paramount priority for a considerable percentage. A majority, totaling 59.1%, adheres to safety protocols and refrains from reusing masks, thereby demonstrating a steadfast commitment to safety guidelines. Most healthcare workers, accounting for 84.1%, opt to stay at home during quarantine, aligning their actions with recommended guidelines aimed at curbing the spread of the virus.

## Discussion

In the wake of the COVID-19 pandemic, healthcare providers worldwide have faced unprecedented challenges. This study delves into the unique perspective of healthcare providers in a developing country, shedding light on their knowledge, attitudes, anxiety levels, and preventive behaviors concerning COVID-19. With 1150 participants, this research offers fresh insights, challenging some previously held assumptions.

Traditionally, studies in this domain have showcased male dominance among participants. However, in this study, we observed a notable shift, with 78.3% of participants being female and 21.7% male. These individuals primarily fell within the age range of 23 to 27 years and hailed from urban areas, where knowledge is diverse, opportunities abound, and COVID-19 awareness is high. Notably, the majority of the participants were physicians, and most had accumulated at least one year of experience in the healthcare sector [2].

In contrast to earlier studies that reported average knowledge levels of a mere 28.14%, our findings provide a more optimistic outlook. In this study, we found that an impressive 80% of healthcare professionals displayed a high level of awareness regarding COVID-19. This shift in knowledge levels suggests that healthcare providers in this developing country are better equipped to tackle the pandemic, potentially leading to improved patient care and safety [10].

Despite global efforts to alleviate social anxiety and panic among healthcare professionals during the pandemic, our study indicated that none of the measures implemented in this developing country appeared to effectively address these concerns. This lack of governmental support and intervention raises questions about the overall well-being of healthcare providers, necessitating further investigation and action [13].

Intriguingly, our study unveiled a stark contrast in anxiety levels between healthcare professionals working in COVID-specific wards and those in general wards. Healthcare providers with direct exposure to COVID-19 patients reported higher levels of anxiety and depression [14]. This finding underscores the need for targeted mental health support and resources for this subset of professionals, who face heightened stressors due to their roles on the frontlines of the battle against the virus.

Drawing comparisons with earlier studies, we discerned interesting patterns related to anxiety and depression among healthcare providers. Contrary to one study, which found anxiety to be mild, our study painted a more alarming picture, indicating severe anxiety and depression levels [8]. This concerning trend highlights the urgency of addressing the mental health of healthcare providers, as their resilience and well-being are paramount to managing the ongoing crisis effectively.

Our study also explored gender-based disparities in anxiety and depression among healthcare providers. We found that females exhibited higher levels of anxiety and depression [12]. This could be attributed to their heightened concern for personal safety and well-being, as well as potential societal pressures. Addressing these disparities and providing tailored support for female healthcare providers is essential to ensure their overall mental and emotional health [15].

Nurses, a critical component of the healthcare workforce, were found to be particularly susceptible to anxiety and depression [2,14]. Their extensive working hours, close patient contact, and the emotional toll of witnessing the effects of COVID-19 on patients may contribute to their vulnerability. Recognizing the unique challenges faced by nurses and implementing strategies to mitigate these challenges is crucial to maintaining a resilient healthcare workforce [5].

COVID-19 has had far-reaching effects on individuals' personal and social lives, and healthcare workers have not been immune to these impacts [6]. Our study highlights that healthcare workers, in particular, have borne a substantial burden, given the increased risk they face due to their profession. As such, it is imperative to consider their unique needs and provide support to mitigate the personal and social consequences of the pandemic.

Study limitations

Firstly, access to the internet and digital devices may not be uniform among participants, potentially leading to selection bias. Secondly, the self-report nature of the survey may introduce response bias, as participants might provide socially desirable answers or inaccurately recall information. Lastly, the study's findings may not be generalizable to all healthcare providers in the country, as the sample may not fully represent this diverse group.

## Conclusions

This study embarks on a transformative journey, delving deep into the experiences of healthcare providers in a developing nation during the COVID-19 era. It goes beyond conventional boundaries, revealing shifting demographics, uncovering valuable insights, and highlighting concerning mental health trends. These findings call for innovative, targeted interventions and robust support systems for our healthcare heroes. In essence, this research paints a vivid picture of the challenges faced by healthcare providers during the pandemic. It underscores gender disparities, recognizes nurses as unsung heroes, and critiques government responses. To navigate this evolving landscape, a new paradigm is needed. Healthcare provider well-being must be integrated into resilient healthcare systems. To bridge knowledge gaps, we advocate for a comprehensive approach. This includes screening, contact tracing, and skill development, transforming personal protective equipment into a well-orchestrated defense. We emphasize institutional education, standardization, and mindfulness, promoting knowledge enhancement through small-group symposiums. These efforts, at individual and institutional levels, lay the foundation for effective government action. Together, we can create a masterpiece of healthcare resilience.

## Appendices

**Table 3:** Questionnaire

QUESTIONNAIRE	
Knowledge, Attitudes, Anxiety, and Preventive Behaviors Related to COVID-19 Among Healthcare Providers: A Developing Country’s Perspective	
Licensed doctors, pharmacists, nurses, paramedical staff, and medical lab technologists are expected to fill in the feedback and give reviews where appropriate. The purpose of this exercise is to collect data and use this information to treat COVID-19 patients more effectively and use preventive measures more effectively.	
Demographic data	Date:
What is your gender?	Male
	Female
What is your age?	23-27
	28-32
	33-37
	38-42
	43 and above
In which health care domain, you are working as:	Physician
	Pharmacist
	Nurses
	Paramedical staff
	Medical lab technologist
For how long have you worked in your profession?	Less than 1 month
	At least 1 month, but less than 3 months
	At least 3 months, but less than 12 months
	At least 1 year, but less than 3 years
	At least 6 years, but less than 10 years
	Ten years or more
Locality	Urban
	Rural
Knowledge towards COVID-19	
The main clinical symptoms of COVID-19 are: (Tick all that apply)	Cough
	Fever
	Sore throat
	Runny nose
	Myalgia
	Diarrhoea
	Difficulty in breathing
Loss of taste and smell are also the feature of COVID-19 infection:	Yes
	No
	Don’t Know
There is currently no effective cure for COVID-19, but only symptomatic and supportive treatment can help most patients recover from the infection:	Yes
	No
	Don’t Know
Are antibiotics effective against coronavirus?	Yes
	No
	Don’t Know
It is not necessary for children and adults to take measures to prevent the infection by the covid-19 virus.	Yes
	No
	Don’t Know
Persons with COVID-19 cannot transmit the virus to others when fever is not present:	Yes
	No
	Don’t Know
Covid-19 virus spreads via respiratory droplets:	Yes
	No
	Don’t Know
It takes 14 days for corona virus symptoms to appear:	Yes
	No
	Don’t Know
Majority of COVID-19 infective patient will not develop illness but elderly patient having chronic illness, DM, COPD, PNEUMONIA is likely to develop severe illness.	Yes
	No
	Don’t Know
Health care professional with direct contact should take tablet hydroxychloroquine as a prophylaxis.	Yes
	No
	Don’t Know
Attitude towards COVID-19	
Are you currently able to test your patients for COVID-19 quickly and easily?	Yes
	No
	Don’t Know
How many patients have you treated with possible COVID-19 symptoms, but have not been able to test for COVID-19?	0-5 patients
	6-10 patients
	11-15 patients
	More than 15
Are you concerned about patients avoiding testing or treatment due to financial or health insurance barriers?	Yes
	No
	Don’t Know
Has the government taken appropriate measures to support the medical supply chain and ensure that your hospital/clinic has the medical supplies necessary to address the COVID- 19 pandemic?	Yes
	No
	Don’t Know
In response to COVID-19, have you increased your use of telemedicine technologies in your clinical practice?	Yes, I have started seeing patients virtually
	No, we have not
	We’re planning to
Among elderly, children, immuno-compromised and adults who do you think exhibit a high complication rate? (Tick all that apply)	Elderly
	Children
	Adults
	Immune-compromised individuals
Anxiety towards COVID-19	
How frequently do you wash your hands?	Every 15 minutes and when required
	Every 30 minutes and when required
	Every 1 hour and when required
	As and when required
Do you feel anxious in gatherings due to COVID-19?	Yes
	No
	Don’t Know
Do you think that following COVID-19 SOP’s is leading to OCD (Obsessive Compulsive Disorder)?	Yes
	No
	Don’t Know
Do you take antidepressants due to this pandemic situation?	Yes
	No
	Don’t Know
In this pandemic, do health care professionals need mental assistance:	Yes
	No
	Don’t Know
Are you anxious about following patients with COVID‐19?	Yes
	No
	Don’t Know
Worried about being infected?	Yes
	No
	Don’t Know
Strict self-protection against covid-19?	Yes
	No
	Don’t Know
Does COVID-19 have impact on your personal life?	Yes
	No
	Don’t Know
Have you been ostracized?	Yes
	No
	Don’t Know
Due to long working hours and attending ample of patients, do you feel depressed/anxious?	Yes
	No
	Don’t Know
Do you feel anxious in following all SOP’s around COVID-19 patients?	Yes
	No
	Don’t Know
Do you have fear of lack of medication and uncontrolled viral spread?	Yes
	No
	Don’t Know
Are you anxious in counselling patients against non-compliance to the COVID-19 vaccine?	Yes
	No
	Don’t Know
Does scarcity of ventilators give you depression?	Yes
	No
	Don’t Know
Behaviours against COVID-19	
Have you been practicing social distancing?	Yes
	No
	Don’t Know
Do you use other workers’ phones, desks and tools?	Yes
	No
	Don’t Know
Do you reuse a mask?	Yes
	No
	Don’t Know
Do you prefer to stay at home during the quarantine?	Yes
	No
	Don’t Know
